# An Unusual Case of Acute Chondrocalcinosis in Wrist Joint Presenting as Cellulitis

**DOI:** 10.7759/cureus.1916

**Published:** 2017-12-06

**Authors:** Razia Awan, Haider Ghazanfar, Karen A Martes Pena, Rafi I Farkhad

**Affiliations:** 1 Geriatric Care, Newark Beth Israel Medical Center; 2 Internal Medicine, Newark Beth Israel Medical Center

**Keywords:** acute chondrocalcinosis, cellulitis, wrist joint, pseudogout, calcium pyrophosphate deposition

## Abstract

Chondrocalcinosis is a common arthritic disorder among elderly patients. We present a case of a 71-year-old woman presenting with an acute episode of the left forearm with hand swelling. A provisional diagnosis of cellulitis was made and the patient was started on intravenous antibiotics. The patient’s condition did not improve. Joint aspiration of the wrist joint was done and showed positive birefringent rhomboid-shaped crystals. A final diagnosis of acute chondrocalcinosis was made.

## Introduction

Calcium pyrophosphate dehydrate (CPPD) deposition can lead to chondrocalcinosis—a common arthritic disorder found among the elderly population. Chondrocalcinosis prevalence in the general population is around 5% [1]. The prevalence of chondrocalcinosis increases with age and about 30% of adults over the age of 80 years are affected by it [2]. The most common joint affected by this disease is the knee joint, and according to a study in the United Kingdom, the prevalence of knee joint chondrocalcinosis is 7% [3]. In most cases, the patients are asymptomatic, while in 25% of cases, CPPD deposition can lead to acute episodes of pseudogout [4]. About 5% of patients with CPPD deposition develop a chronic rheumatoid arthritis-like condition. The clinical manifestation of chondrocalcinosis can be misleading. In this case report, we present the case of a 71-year-old woman who presented with an acute episode of left forearm swelling.

## Case presentation

A 71-year-old woman with a past medical history of ulcerative colitis for 35 years and polyarthritis for four years presented to the emergency room with left forearm and hand swelling for the past two days. She went to a local primary care doctor who started her on 0.6 mg of colchicine two times a day for two days and then daily, but her symptoms did not improve.

The swelling started suddenly two days prior and progressively worsened with time. Her left forearm and hand swelling were associated with pain, and she was unable to move her arm because of it. The patient graded the pain an eight on of a scale of one to 10. The pain worsened upon compression and on movement of the arm. There were no relieving factors. She also had concerns of a low-grade fever but denied swelling or pain in any other joints of the body.

On physical examination, there was redness and swelling present in her entire left forearm and hand. There was tenderness on palpation of her left forearm and hand. The pain increased significantly on slight pressure. Peripheral pulses were palpable. The sensory and motor system was intact. She had a 100°F temperature. Her pulse was 72 beats per minute and regular in rate, volume, rhythm, and character.

A provisional diagnosis of cellulitis was made. She was admitted to the hospital and was started on intravenous fluid, pain medication, intravenous antibiotics ceftriaxone and vancomycin, intravenous steroids, and colchicine. The patient had an elevated white blood cell count with left shift, an erythrocyte sedimentation rate (ESR) of 82 mm/hr, and a C-reactive protein (CRP) level of 25.04 mg/L (Table 1).

**Table 1:** Laboratory Test Results CRP, C-reactive protein; HCT, hematocrit; MCV, mean corpuscular volume; RBC, red blood cells; WBC, white blood cells

Laboratory Tests	Result
Complete Blood Count	
WBC count	14.2 x 10^3^/µL
RBC count	4.15 x 10^6^/µL
Hemoglobin	11.2 g/dL
HCT	35.2%
MCV	84.6 µm^3^
Platelets	380 10^3^/µL
Differential Count	
Neutrophils	86%
Lymphocytes	5%
Monocytes	9%
Eosinophils	0%
Basophils	0%
Inflammatory Markers	
Erythrocyte sedimentation rate	82 mm/h
C-reactive protein	25.04 mg/L

**Figure 1:**
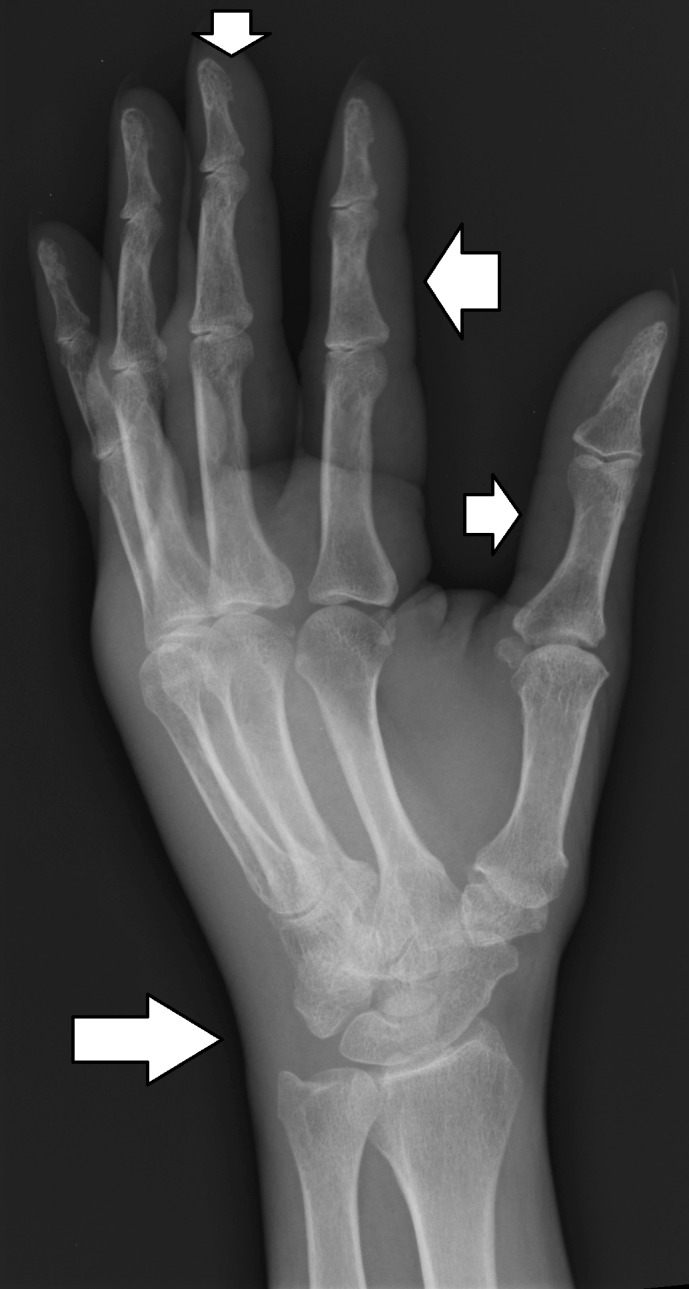
X-ray of the wrist joint showing soft-tissue swelling in the hand and at the wrist joint

The patient’s condition did not improve. X-rays of the shoulder, elbow and wrist joint were done. The x-ray of the wrist joint showed soft tissue swelling in the hand and around the wrist (Figure 1). There was no other abnormality.

Ultrasound of the left forearm showed increased soft tissue swelling. Her uric acid level was normal. Joint aspiration of the wrist joint was done and showed positive birefringent rhomboid-shaped crystals. A final diagnosis of acute chondrocalcinosis was made. The patient was started on non-steroidal anti-inflammatory drugs (NSAIDS) and low dose colchicine. Hourly cool packs for four hours were also advised. The patient’s fever and swelling subsided. The patient was discharged in stable condition. She was advised to follow up in the clinic after one week.

## Discussion

This report demonstrates that CPPD could often be misdiagnosed or mimic another disorder such as cellulitis, as seen in our patient. It is fundamental that CPPD is considered as part of a differential diagnosis for a patient that has symptoms associated with joint pain, erythema, warmth, tenderness, and swelling of any joint. Patients could frequently present with constitutional symptoms, fever, and chills that could last from weeks to months [5]. Pseudogout is a metabolic arthropathy characterized by the presence of calcium pyrophosphate (CPP) crystals located in the periarticular and articular tissues. Diagnosis is definitive by the visualization of positive birefringent rhomboid-shaped crystals in the affected joint synovial fluid [5].

There have been reports of rare cases of acute chondrocalcinosis mimicking infection. Bridges et al. reported a case of pseudogout of the cervical and thoracic spine mimicking an infection after a lumbar fusion [5]. Pseudogout can present with nonspecific laboratory results that suggest bacterial infection and inflammatory symptoms [6]. In our case, high values for white blood cells, CRP, and ESR associated with our patient’s clinical signs and symptoms indicated cellulitis. This demonstrates that pseudogout should always be considered in the differential diagnosis to provide prompt and adequate patient care.

Patients with CPPD can have negative x-rays. Forty percent of the patients with CPPD may have no radiological findings [7]. The identification of CPP crystals in synovial fluid by microscopy is the standard confirmatory test for the diagnosis of CPPD [6].

Patients with asymptomatic CPPD do not require any treatment [8]. Medication used to manage a patient with an acute attack include cool packs, NSAIDS, and low dose colchicine. Joint aspiration with intraarticular long-acting glucocorticoid injection might be needed if previous medications do not help in relieving the symptoms [8]. In some patients, a short trial of oral or parenteral glucocorticoids can be given.

## Conclusions

Chondrocalcinosis is a common arthritic disorder among elderly patients. The prevalence of chondrocalcinosis increases with age. Our patient was a 71-year old woman who presented with acute chondrocalcinosis. Although acute chondrocalcinosis is a rare presentation, physicians should keep chondrocalcinosis as a possible differential diagnosis for a patient presenting with sudden onset swelling of the arm.
